# The activation of cGAS‐STING pathway causes abnormal uterine receptivity in aged mice

**DOI:** 10.1111/acel.14303

**Published:** 2024-08-07

**Authors:** Si‐Ting Chen, Wen‐Wen Shi, Feng Ran, Cheng‐Kan Liu, Hui‐Na Luo, Li‐Juan Wu, Ying Wu, Tong‐Tong Zhang, Zeng‐Ming Yang

**Affiliations:** ^1^ Key Laboratory of Plateau Mountain Animal Genetics, Breeding and Reproduction, Ministry of Education Guizhou University Guiyang China; ^2^ College of Animal Science Guizhou University Guiyang China; ^3^ College of Veterinary Medicine South China Agricultural University Guangzhou China

**Keywords:** aging, CD14, cGAS‐STING, progerin, uterine receptivity

## Abstract

Maternal age is one of the most important factors affecting the success of maternal pregnancy. Uterine aging is the leading cause of pregnancy failure in older women. However, how uterine aging affects uterine receptivity and decidualization is unclear. In this study, naturally aged one‐year‐old female mice were used to investigate effects of maternal age on embryo implantation during early pregnancy. In our study, we found abnormal uterine receptivity in aged mice. Aged mouse uterus indicates a decrease in nuclear LAMIN A, and an increase in PRELAMIN A and PROGERIN. In aged mouse uterus, double‐stranded DNA (dsDNA) in cytoplasmic fraction is significantly increased. PROGERIN overexpression in mouse uterine epithelial cells and epithelial organoids leads to nuclear DNA leakage and impaired uterine receptivity. DNase I, DNase II, and TREX1 are obviously reduced in aged mouse uterus. Treatments with foreign DNA or STING agonist significantly downregulate uterine receptivity markers and activate cGAS‐STING pathway. Uterine estrogen (E_2_) concentration is significantly increased in aged mice. After ovariectomized mice are treated with a high level of E_2_, there are significant increase of PROGERIN and cytoplasmic DNA, and activation of cGAS‐STING pathway. CD14 is significantly increased in aged uterus. Intrauterine CD14 injection inhibits embryo implantation. In vitro CD14 treatment of cultured epithelial cells or epithelial organoids decreases uterine receptivity. Uterine abnormality in aged mouse can be partially rescued by STING inhibitor. In conclusion, uterine PROGERIN increase in aged mouse uterus results in cytoplasmic DNA accumulation and cGAS‐STING pathway activation. CD14 secretion in aged uterus impairs uterine receptivity.

## Graphical abstract

The high level of uterine E_2_ impairs uterine receptivity in aged mice. The increase of PRELAMIN A, PROGERIN and cytoplasmic DNA in aged mouse uterus leads to activation of cGAS‐STING pathway. CD14 accumulation derived from activated cGAS‐STING causes abnormal receptivity.

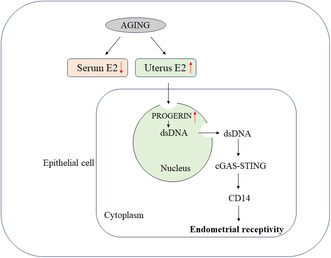

**Abbreviations**
AREG: Amphiregulin
C3: Complement C3
cGAS: Cyclic GMP–AMP synthase
COX2: Cyclooxygenase 2
ds DNA: Double‐stranded DNA
E_2_
: Estrogen
Hand2: Heart and neural crest derivatives expressed 2
HOXA10: Homeobox A10
IFN: Interferon
Ihh: Indian hedgehog
IRF3: IFN regulatory factor 3
LTF: Lactoferin
MUC1: Mucin1
MSX1: Msh homeobox 1
P4: Progesterone
SA‐β‐GAL: Senescence‐associated beta‐galactosidase
SDNA: Salmon sperm DNA
STAT3: Signal transducer and activator of transcription 3
STING: Stimulator of interferon genes
TBK1: TANK‐binding kinase 1
TREX1: Three‐prime repair exonuclease 1

## 1 INTRODUCTION

Maternal age is the most critical factor impacting reproductive success. Female fertility in humans begins to drop in the early thirties and typically reaches zero by the age of 50 (Levitis et al., 2013). Older women and female mammals have irregular menstrual or estrous cycles, ovulation problems, lower fertility, and lower conception rates (Dunson et al., 2004). With the increase of maternal age, caesarean section, instrumental delivery, and pregnancy complications are also on the rise (Patel et al., 2017). Conception rate in aged females is also significantly low (Li et al., 2017). Accumulating evidence suggests that the main causes of age‐related infertility are a loss in ovarian reserve function, and a decline in oocyte quality and embryonic development capacity (Cimadomo et al., 2018). Ovarian aging is associated with a considerable decrease in oocyte reserve and oocyte quality (Tatone et al., 2008). The donated eggs from younger women can assist older women with low pregnancy rates during in vitro fertilization (IVF) (Templeton et al., 1996). Therefore, the decrease in oocyte quality and quantity is a significant characteristic of reproductive age.

Uterine aging is previously considered to have no influence on IVF outcomes (Navot et al., 1994). However, there are conflicting data on the effect of uterine age on pregnancy rates (Noyes et al., 2001). A successful pregnancy is dependent on the mother's physiological state because embryonic growth is significantly influenced by the maternal environment (Fleming et al., 2018). The number of advanced maternal age (AMA) women who choose assisted reproductive technology (ART) also increases. However, AMA often leads to lower ART successful rate (Vitagliano et al., 2023). This indicates that uterine aging is an important factor affecting fertility, reproductive health, and uterine diseases (Wu et al., 2023). The decline in uterine function with aging may be linked to endometrial hormonal dysregulation and reduced endometrial receptivity (Wu et al., 2023; Zhao et al., 2023). Inflammation, fibrosis, and cellular senescence are involved in uterine aging (Pathare et al., 2023). The aging uterus may lead to increased embryo loss and decreased embryo development capacity at the implantation stage due to vascular dysfunction, impaired decidual response, and decreased uterine prostaglandin synthesis (Morton et al., 2017). The hormonal response is attenuated in an aged uterus (Woods et al., 2017). Aged mice show an impairment of the artificially induced decidual response (Shapiro & Talbert, 1974). Premature endometrial senescence is linked to poor reproductive outcome. Senescence of endometrial stromal cells results in poor endometrial decidualization and embryo implantation failure (Deryabin & Borodkina, 2022). Moreover, defective uterine decidualization results in preeclampsia, infertility, and endometriosis (Marquardt et al., 2019). Therefore, endometrial aging affects the implantation rate, clinical success rate, and live birth rate in elderly women (Pathare et al., 2023).

Senescence is an irreversible form of long‐term cell cycle arrest caused by excessive intracellular or extracellular stress or injury. Senescence is characteristic by an increase of senescence‐associated beta‐galactosidase (SA‐β‐GAL), P16INK4a, p21CIP1, and P53 (Calcinotto & Alimonti, 2017). Lamina is a network of atypical intermediate filaments located beneath the inner membrane of the nuclear envelope and composed of mature LAMIN A, LAMIN B1, LAMIN B2, and LAMIN C. LAMIN A is specifically implicated in maintaining the structural integrity of the nucleus (Turgay & Medalia, 2017). The nuclei of senescent cells often show laminar breakdown, downregulated expression of LAMIN B (Freund & Campisi, 2012), and an increase of immature form of LAMIN A (PRELAMIN A) or PROGERIN (Lenain et al., 2017). Polytransmembrane enzyme zinc metalloproteinase (ZMPSTE24) is unable to cleave the farnesylated and carboxyl methylated LAMIN A. A point‐mutation in the LAMIN A gene results in accumulation of the truncated premature protein PROGERIN in the nuclear periphery (Scaffidi & Misteli, 2006). PROGERIN accumulation leads to abnormal nuclear morphology and nuclear structural instability (Danielsson et al., 2022).

Proper inflammation is important for embryo implantation, decidualization, and delivery (Nadeau‐Vallée et al., 2016). At human implantation site, inflammation factors are transiently increased (Zhao et al., 2021). Local damage in uterine lumen can stimulate decidual formation in pseudopregnant rodents (Dekel et al., 2009). In IVF patients, endometrial scratch before oocyte retrieval also increases implantation and pregnancy success (Mor et al., 2011). Natural aging is characterized by a disrupted balance between proinflammatory and anti‐inflammatory mediators (Tan et al., 2023). Senescent cells release senescence‐associated secretory phenotype (SASP) factors to impair tissue homeostasis and to promote inflammation (Coppé et al., 2010). The accumulation of senescent cells can drive chronic inflammation (Roth‐Walter et al., 2023). The low level of immune activation leads to the local lack of angiogenic activity and pregnancy failure. Uterine curettage in the already inflamed endometrium may cause excessive inflammation of the endometrium and lead to implantation failure (Rahmati & Lédée, 2020). Ageing is followed by micronuclear formation and cytoplasmic DNA accumulation, which will lead to the activation of the cyclic GMP‐AMP synthase (cGAS) stimulator of interferon genes (STING) pathway (Li & Chen, 2018). Microglia can enter aging‐associated state once the cGAS‐STING pathway is activated (Gulen et al., 2023).

The cGAS‐STING signaling is the major signaling pathway for sensing foreign DNA and eliciting an effective immune response, involving in autoimmunity and sterile inflammation (Hopfner & Hornung, 2020). cGAS senses foreign double‐stranded DNAs (dsDNA) to generate cGAMP. cGAMP as a second messenger activates STING and TBK1 (Sun & Hornung, 2022). TBK1 phosphorylates IRF3 to induce IRF3 translocation into nucleus for stimulating genes encoding for type I interferons (Hopfner & Hornung, 2020). cGAS is also involved in cellular senescence (Yang et al., 2017). PROGERIN‐induced genomic instability is associated with replication stress and activation of the cGAS‐STING pathway (Coll‐Bonfill & Gonzalo, 2020). In health cells, DNA is mostly enclosed in the nucleus and mitochondria. Extracellular or cellular DNase enzymes can digest extracellular or cytoplasmic dsDNA to avoid activating cGAS‐STING pathway, including extracellularly DNase I, phagolysosomal compartment DNase II, and cytoplasmic exonuclease 1 (TREX1, also known as DNase III) (Kawane et al., 2014). Cells infected with a virus or bacteria, cancer cells and senescent cells are often filled with cytoplasmic dsDNA due to cell shrinkage, chromatin condensation, or membrane blebbing (Du et al., 2023). cGAS‐STING pathway has emerged as an essential factor for driving inflammation‐induced tumor growth (Ahn et al., 2014). However, the underlying mechanism how cGAS‐STING pathway is involved in early pregnancy and aging are still unclear.

Our previous study has shown that the pregnancy rate and implantation number in 12‐month‐old mice significantly decline because of the low ovulation rate and steroid hormonal secretion in aging mice (Li et al., 2017). In this study, a one‐year‐old mouse model of natural aging was used. Our data indicated that the increase of uterine PROGERIN in aged mice on day 4 of pregnancy leads to the accumulation of cytoplasmic DNA and activation of cGAS‐STING pathway, causing the secretion of monocyte differentiation antigen CD14 and impairment of the uterine receptivity.

## 2 RESULTS

### 2.1 Abnormal uterine receptivity in aged mice

**FIGURE 1:**
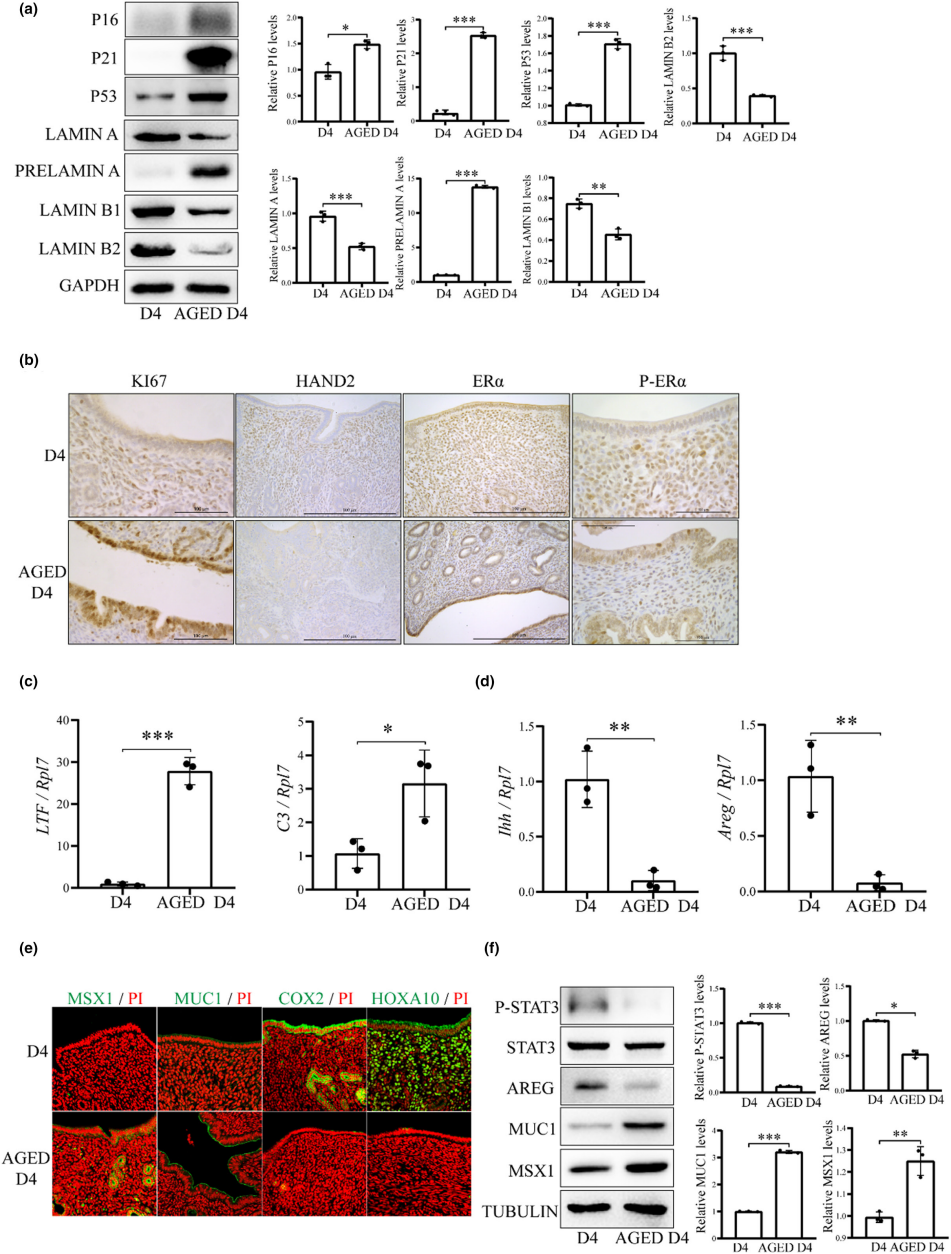
The receptivity‐related changes in the uteri of control mice (D4) and aged mice (Aged D4) on day 4 of pregnancy. (a) Western blot analysis of P16, P21, P53, LAMIN A, PRELAMIN A, LAMIN B1 and LAMIN B2 protein levels. (b) Immunohistochemistry of KI67, HAND2, ERα and p‐ERα. Scale bar, 100 μm. (c) QPCR analysis on the mRNA levels of estrogen target genes (*Ltf* and *C3*). (d) QPCR analysis on the mRNA levels of progesterone‐targeted genes (*Ihh* and *Areg*). (e) Immunofluorescence of COX2, MUC1, MSX1, and HOXA10. Scale bar, 100 μm. (f) Western blot analysis of P‐STAT3, STAT3, AREG, MUC1, and MSX1 protein levels (*N* = 4 mice). *p < 0.05; **p < 0.01; ***p < 0.001.

Increased SA‐beta‐gal activity, reduced LAMIN A and increased PRELAMIN A are the markers of aging (Freund et al., 2012). The protein levels of LAMIN A, LAMIN B1 and LAMIN B2 were significantly decreased in aged mice compared with young mice. However, the level of PRELAMIN A, a defective and immature form of LAMIN A (Lenain et al., 2017), was significantly increased in aged mice (Figure 1a). This suggested that senescence obvious occurred in the uterus of one‐year‐old mice.

Embryo implantation window is regulated by the balanced progesterone (P_4_) and estrogen (E_2_) signaling (Hewitt & Korach, 2011). Compared with young mice, the uterine epithelium of aged mice showed an increase in estrogen receptor (ERα), p‐ERα and cell proliferation marker KI67 immunostaining (Figure 1b). Lactoferin (LTF) and complement C3 (C3) are target genes of E_2_ signaling (Hantak et al., 2014; Sundstrom et al., 1989). The levels of *Ltf* and *C3* mRNA expression were significantly increased in the uterus of aged mice compared with young mice (Figure 1c). These results suggested that there was an increase of E_2_ activity and uterine epithelial cell proliferation in aged mouse uterus. Heart and neural crest derivatives expressed 2 (HAND2) and Indian hedgehog (Ihh) are the P_4_‐stimulated genes (Hewitt & Korach, 2011). HAND2 immunostaining and *Ihh* mRNA level were decreased in aged mice (Figure 1b,d). Cyclooxygenase 2 (COX2), homeobox A10 (HOXA10), amphiregulin (AREG) and signal transducer and activator of transcription 3 (STAT3) phosphorylation are recognized markers for mouse uterine receptivity (Hantak et al., 2014; Hu et al., 2023). In this study, the immunofluorescence levels of COX2 and HOXA10 were significantly downregulated in aged mouse uterus (Figure 1e). The protein levels of p‐STAT3 and AREG were significantly decreased in aged mouse uterus (Figure 1f). Mucin 1 (MUC1) and Msh homeobox 1 (MSX1) are considered as the markers of endometrial nonreceptivity (Hantak et al., 2014). Both the immunofluorescence and protein levels of MUC1 and MSX1 were significantly increased in aged mouse uterus (Figure 1e,f). These results suggested that the uterine receptivity was impaired in aged mouse uterus.

### 2.2 PROGERIN and cytosolic DNA accumulation in aged mouse uterus

**FIGURE 2:**
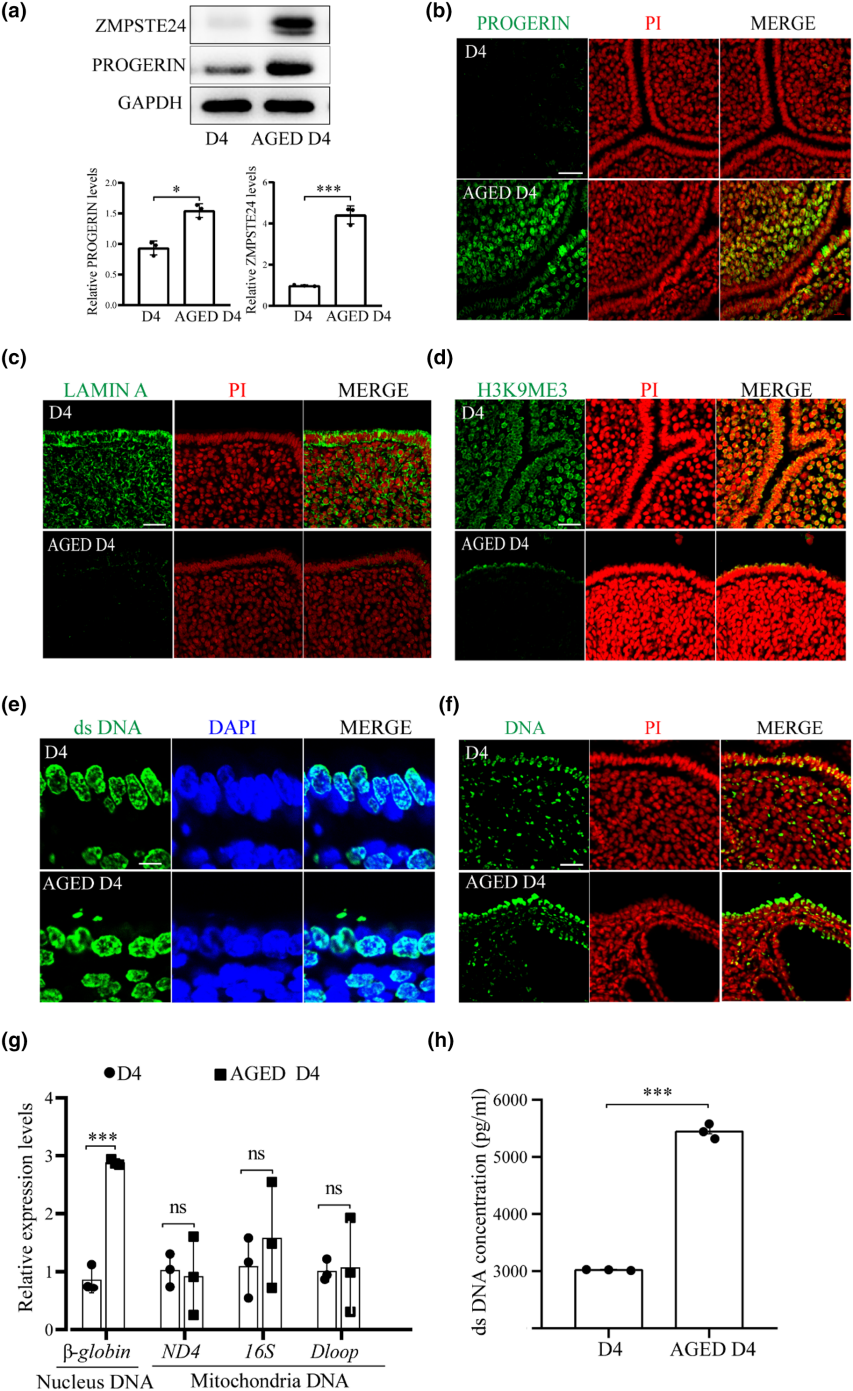
The LAMIN‐related changes and cytoplasmic DNA in the uteri of control mice (D4) and aged mice (Aged D4) on day 4 of pregnancy. (a) Western blot analysis of ZMPSTE24 and PROGERIN levels. (b) PROGERIN immunofluorescence of in young and aged mouse uteri on D4. Scale bar, 50 μm. (c) LAMIN A immunofluorescence. Scale bar, 50 μm. (d) H3K9ME3 immunofluorescence. Scale bar, 50 μm. (e) dsDNA immunofluorescence with anti‐dsDNA lgG. Scale bar, 50 μm. (f) dsDNA immunofluorescence with anti‐dsDNA lgM. Scale bar, 50 μm. (g) QPCR analysis on the levels of nuclear DNA (*β‐globin*) and mitochondria DNAs (*Nd4*, *16S*, and *Dloop*) in the cytoplasmic fraction. (h) The PICOGREEN‐measured DNA concentration in the cytoplasmic fraction (*N* = 5 mice). ns, not significant; *p < 0.05; ***p < 0.001.

Splicing mutation in LAMIN A results in PROGERIN or ∆50 PRELAMIN A, an internally deleted PRELAMIN A variant. PROGERIN retains its CAAX motif, but lacks the ZMPSTE24 cleavage site (Scaffidi & Misteli, 2006). The protein levels of PROGERIN and ZMPSTE24 were significantly increased in aged mouse uterus (Figure 2a). PROGERIN immunofluorescence was also increased in aged mouse uterus (Figure 2b). LAMIN A is essential to the integrity of nuclear envelope (Turgay & Medalia, 2017). LAMIN A immunofluorescence was significantly reduced in aged mouse uterus (Figure 2c). H3K9ME3 is a repressive chromatin marker (Guelen et al., 2008). H3K9ME3 immunofluorescence was also reduced in aged mouse uterus (Figure 2d), suggesting that the heterochromatin in the aged mouse uterus was in an unstable state.

PROGERIN accumulation in the nuclear envelope can cause abnormal nuclear shape and leak of nuclear DNA (Yang et al., 2005). DNA concentration measured through immunofluorescence of DNA and PICOGREEN also showed a significant increase in aged mouse uterus compared with young mice and in uterine epithelial cells, there was extranuclear dsDNA in aged mouse uterus (Figure 2e,f). By cell fraction, the nuclear dsDNA levels (*β‐globin*) (Ueda et al., 2023) in cytoplasmic fraction were also increased in aged mouse uterus, but mitochondrial DNA levels (*Nd4*, *16S*, and *Dloop*) (Antón & Traba, 2022) didn't show an obvious change (Figure 2g). By examining the cell fraction, it was found that the concentration of ds DNA in the uterus of the aged mice was significantly higher than that of the young mice (Figure 2h). These results suggested that PROGERIN increase in aged mouse uterus might lead to nuclear DNA leakage and cytosolic DNA accumulation.

### 2.3 cGAS‐STING activation in aged mouse uterus

**FIGURE 3:**
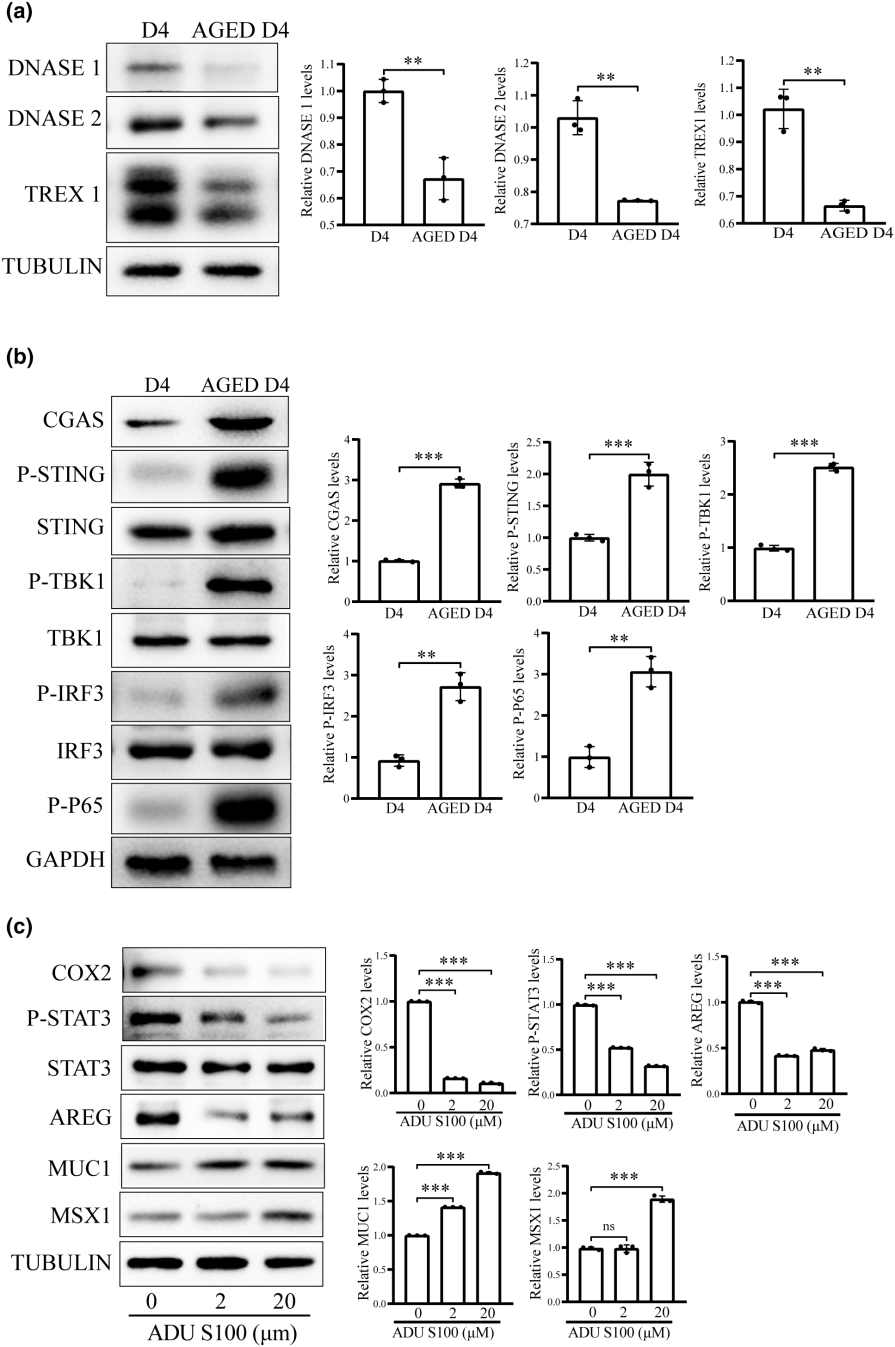
The cGAS‐STING‐related changes and effects of STING agonist on uterine receptivity in the uteri of control mice (D4) and aged mice (Aged D4) on day 4 of pregnancy. (a) Western blot analysis of DNase I, DNase II and TREX1 protein levels. (b) Western blot analysis of the cGAS‐STING‐related proteins. (c) Western blot analysis of the receptivity‐related proteins after uterine epithelial organoids were treated with ADU S100 (STING agonist) for 48 h. ns, not significant; **p < 0.01; ***p < 0.001.

There are three kinds of DNases, DNase I, DNase II, and TREX1 (Motwani & Fitzgerald, 2019). The protein levels of DNase1, DNase2, and TREX1 were significantly reduced in aged mouse uterus (Figure 3a). Moreover, the protein levels of cGAS, P‐STING, P‐TBK1 and P‐IRF3 were all significantly upregulated in aged mouse uterus (Figure 3b). Treatment of mouse epithelial organoids with 20 μM ADU S100, an activator of STING, significantly reduced the protein levels of uterine receptivity markers (COX2, P‐STAT3, and AREG), while increased the protein levels of uterine nonreceptivity markers (MUC1 and MSX1) (Figure 3). These results suggested that cytosolic accumulation of dsDNA could activate the cGAS‐STING pathway and impair uterine receptivity.

### 2.4 PROGERIN overexpression leads to cytoplasmic DNA accumulation and cGAS‐STING pathway activation

**FIGURE 4:**
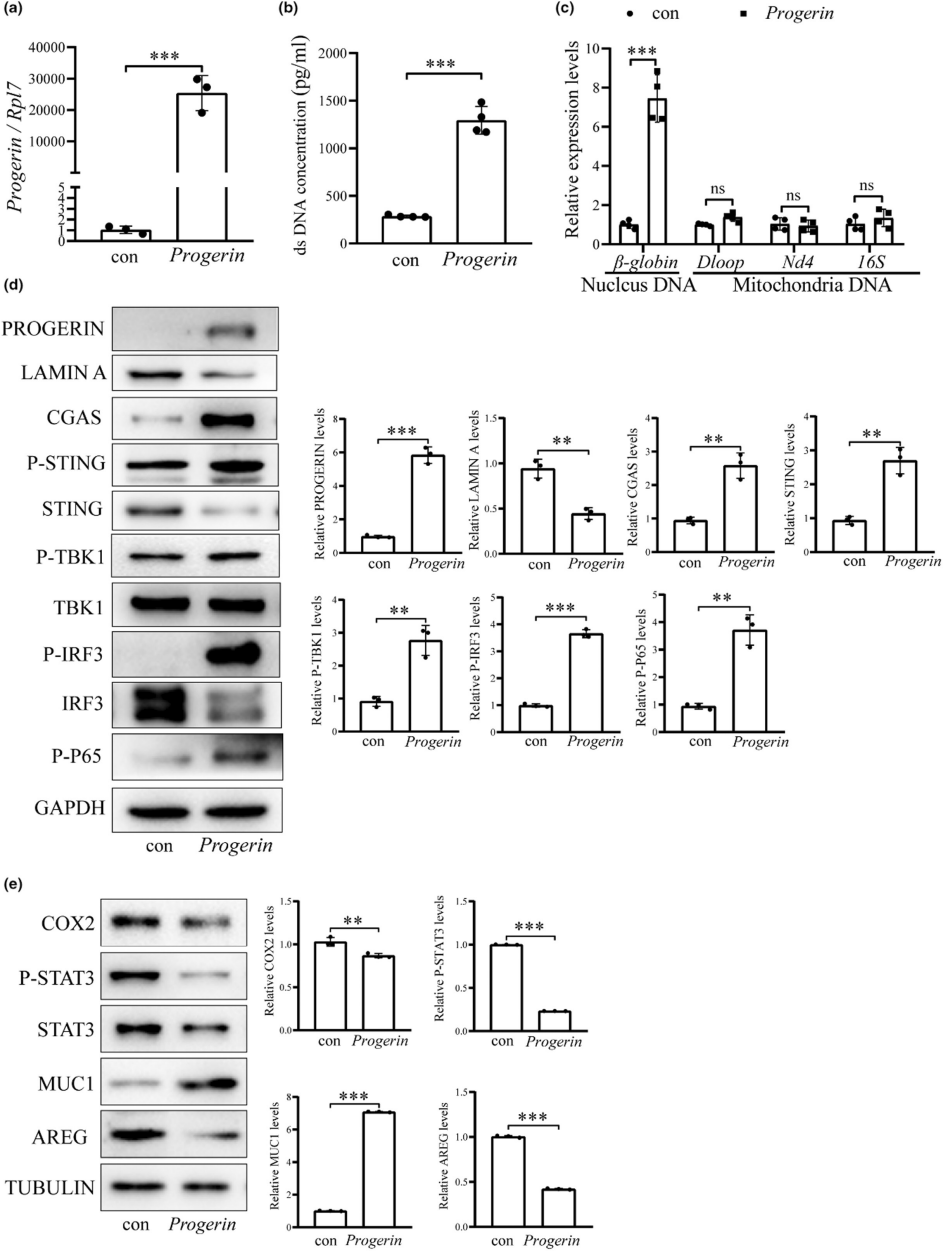
PROGERIN overexpression leads to cytoplasmic DNA accumulation and cGAS‐STING pathway activation. (a) QPCR analysis of *Progerin* mRNA level when *Progerin* gene was overexpressed in mouse epithelial cells. (b) The concentration of dsDNA in the cultured medium after *Progerin* gene was overexpressed in mouse epithelial cells. (c) QPCR analysis of nuclear DNA (*β‐globin*) and mitochondrial DNA (*Nd4*, *16S*, and *Dloop*) levels in the cultured medium after *Progerin* gene was overexpressed in mouse epithelial cells. (d) Western blot analysis of PEOGERIN, LAMIN A and cGAS‐STING‐related protein levels after *Progerin* gene was overexpressed in mouse epithelial cells. (e) Western blot analysis of the receptivity‐related protein levels after *Progerin* gene was overexpressed in mouse epithelial organoids. Con, empty vector control; *Progerin*, *Progerin* overexpression. ns, not significant;**p < 0.01; ***p < 0.001.

To verify the role of PROGERIN in the uterus of aged mice, PROGERIN plasmids were transfected into mouse epithelial cells, showing a significant increase of *Progerin* mRNA (Figure 4a). DNA concentration measured by PICOGREEN was also significantly increased in the culture medium collected from *Progerin*‐overexpressed mouse epithelial cells (Figure 4b). By cell fraction, the concentration of nuclear DNA (*β‐globin*) in the cytoplasmic fraction of *Progerin*‐overexpressed mouse epithelial cells was significantly increased, while the concentration of mitochondrial DNA (*16S*, *Nd4*, and *Dloop*) wasn't significantly different (Figure 4c). These results showed that the increase of PROGERIN caused a leakage and cytoplasmic accumulation of nuclear dsDNA. After PROGERIN was overexpressed in epithelial cells, the protein levels of cGAS, STING, P‐TBK1 and P‐IRF3 were significantly increased (Figure 4d). In *Progerin*‐overexpressed epithelial organoids, the protein levels of uterine receptivity markers (COX2, P‐STAT3 and AREG) were significantly decreased, while MUC1 was significantly increased (Figure 4e). These results suggested that increased PROGERIN led to the accumulation of cytosolic DNA and activation of cGAS‐STING pathway.

### 2.5 Exogenous DNA activates the cGAS‐STING pathway and impairs uterine receptivity

**FIGURE 5:**
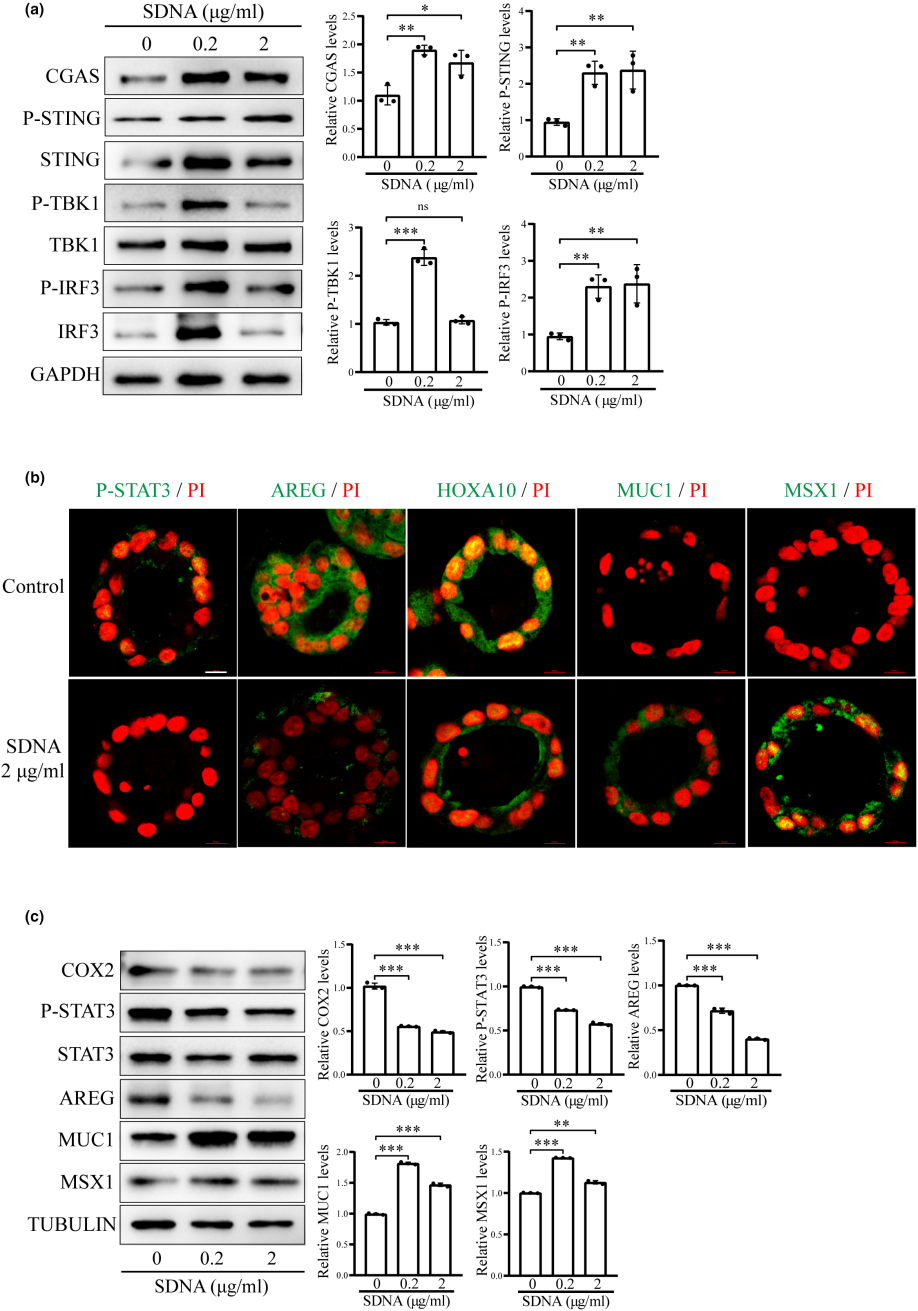
Foreign DNA activates cGAS‐STING pathway and affects uterine receptivity. (a) Western blot analysis of cGAS‐STING‐related protein levels after mouse epithelial cells were transfected with salmon sperm DNA (SDNA) for 48 h. (b) Immunofluorescence of p‐STAT3, AREG, HOXA10, MUC1 and MSX1 after mouse epithelial organoids were transfected treated with SDNA for 48 h. Scale bar, 100 μm. (c) Western blot analysis of receptivity‐related protein levels after mouse epithelial organoids were transfected with SDNA for 48 h. ns, not significant; *p < 0.05; **p < 0.01; ***p < 0.001.

To explore the role of cytoplasmic DNA, mouse epithelial cells were transfected with salmon sperm DNA (SDNA). SDNA significantly upregulated the protein levels of cGAS, p‐STING, p‐TBK1, and p‐IRF3 in mouse epithelial cells (Figure 5a). After mouse epithelial organoids were treated with SDNA, p‐STAT3, AREG, and HOXA10 immunofluorescence was reduced, while MUC1 and MSX1 immunofluorescence was obviously increased (Figure 5b). Western blot data also showed that SDNA significantly downregulated the protein levels of epithelial receptivity markers (COX2, P‐STAT3, HOXA10, and AREG), and increased the protein levels of endometrial nonreceptivity markers (MUC1 and MSX1) (Figure 5c). These results also confirmed that exogenous DNA could activate cGAS‐STING pathway in mouse epithelial cells.

### 2.6 The high level of uterine E_2_ stimulates PROGERIN increase and activation of cGAS‐STING pathway

**FIGURE 6:**
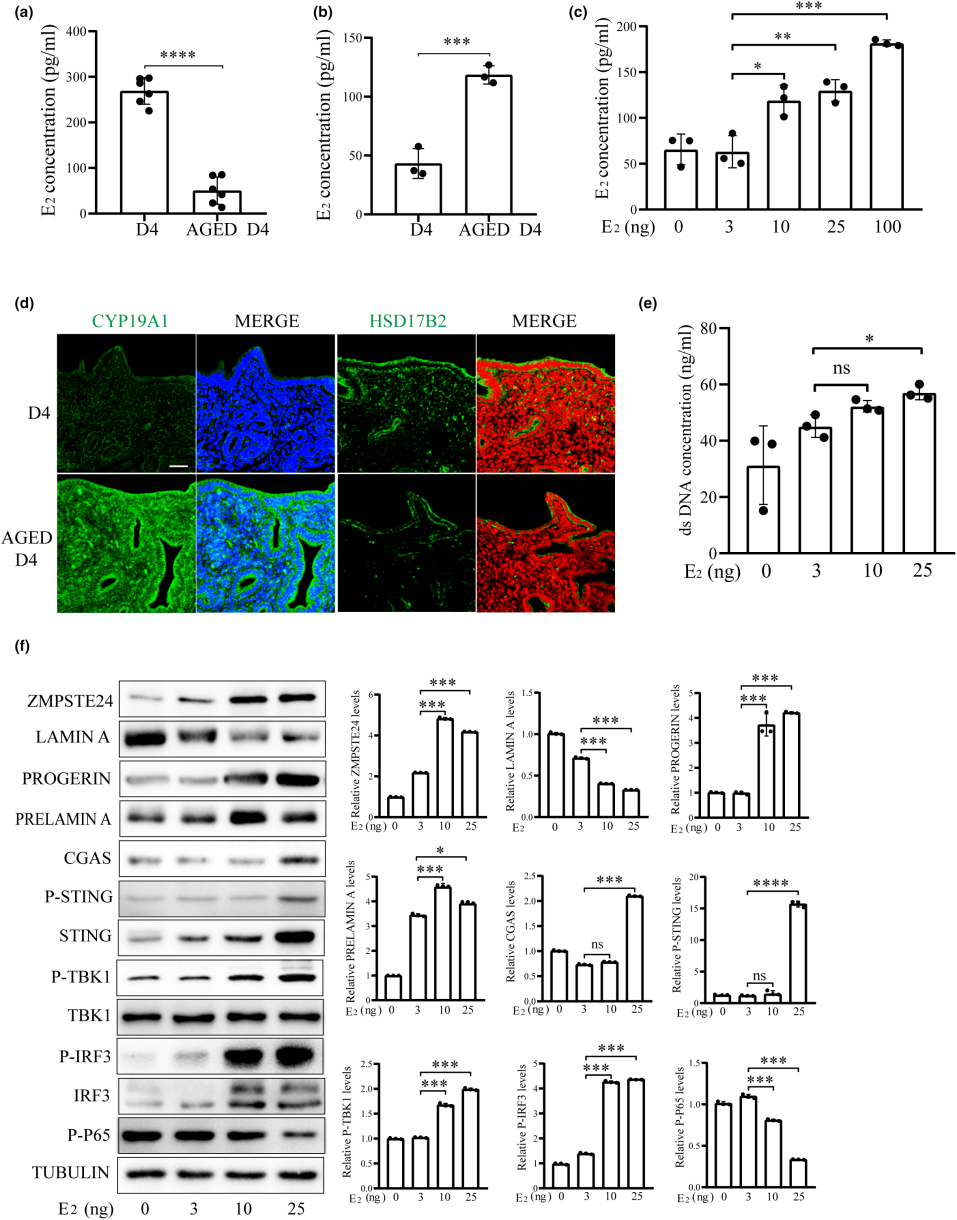
Effects of excess estrogen on PROGERIN and cGAS‐STING pathway in aged mouse uterus. (a) Serum E_2_ concentrations in young (D4) and aged mice (AGED D4) on day 4 of pregnancy. (b) E_2_ concentrations in the uterus of young and aged mice on day 4 of pregnancy. (c) Uterine E_2_ concentration after ovariectomized mice were injected subcutaneously with 0, 3, 10, or 25 ng estradiol‐17β per mouse for 7 days. (d) Immunofluorescence of CYP19A1and HSD17B2 in the uterus of young and aged mice on day 4 of pregnancy. Scale bar, 100 μm. (e) Cytoplasmic dsDNA concentration in the uterus after ovariectomized mice were injected subcutaneously with 0, 3, 10, or 25 ng estradiol‐17β per mouse for 7 days. (f) Western blot analysis of ZMPSTE24, LAMIN A, PROGERIN, PRELAMIN A and cGAS‐STING pathway‐related protein levels in the uterus after ovariectomized mice were injected subcutaneously with 0, 3, 10, or 25 ng estradiol‐17β per mouse for 7 days. ns, not significant; *p < 0.05; **p < 0.01; ***p < 0.001, ****p < 0.0001.

Compared to young mice, serum E_2_ level was decreased, but uterine E_2_ level was increased in aged mice (Figure 6a,b). To explore the action of E_2_ on nuclear envelope and cGAS‐STING pathway, ovariectomized mice were subcutaneously injected with different concentrations of E_2_. Previous study indicated that injection of 3 ng estradiol‐17β per mouse should be close to the physiological concentration of E_2_ in mice (Ma & Dey, 2003). Based on our results, 3 ng estradiol‐17β per mouse was close to E_2_ concentration in young mice, while 10 ng and 25 ng estradiol‐17β per mouse was close to E_2_ level in aged mice (Figure 6c).

Moreover, the protein level of cytochrome P45019A1 (CYP19A1) for E_2_ synthesis (Thomas & Potter, 2013) was significantly increased in aged mouse uterus (Figure 6d), while the protein level of 17β‐hydroxysteroid dehydrogenase type 2 (HSD17B2) for E_2_ metabolism (Rizner, 2009) was significantly decreased in aged mouse uterus (Figure 6d). After cell fractionation, the dsDNA of cytoplasmic components was detected by PICOGREEN analysis. Cytoplasmic DNA concentration in 25 ng estradiol‐17β‐injected mouse uterus was significantly higher than that in 3 ng estradiol‐17β‐injected mice (Figure 6e). Compared to 3 ng estradiol‐17β, LAMIN A protein levels were significantly decreased, while PROGERIN and PRELAMIN A protein levels were significantly increased in the uteri of 10 ng‐or 25 ng estradiol‐17β‐injected mice (Figure 6f). These results suggested that E_2_ could cause an increase of PROGERIN and accumulation of cytosolic DNA. The protein levels of cGAS, P‐STING, P‐TBK1 and P‐IRF3 were also significantly increased in 10 ng or 25 ng estradiol‐17β‐injected mouse uterus (Figure 6f). Taken together, the results suggested that a high level of E_2_ could activate the cGAS‐STING pathway.

### 2.7 CD14 secretion and action on embryo implantation

**FIGURE 7:**
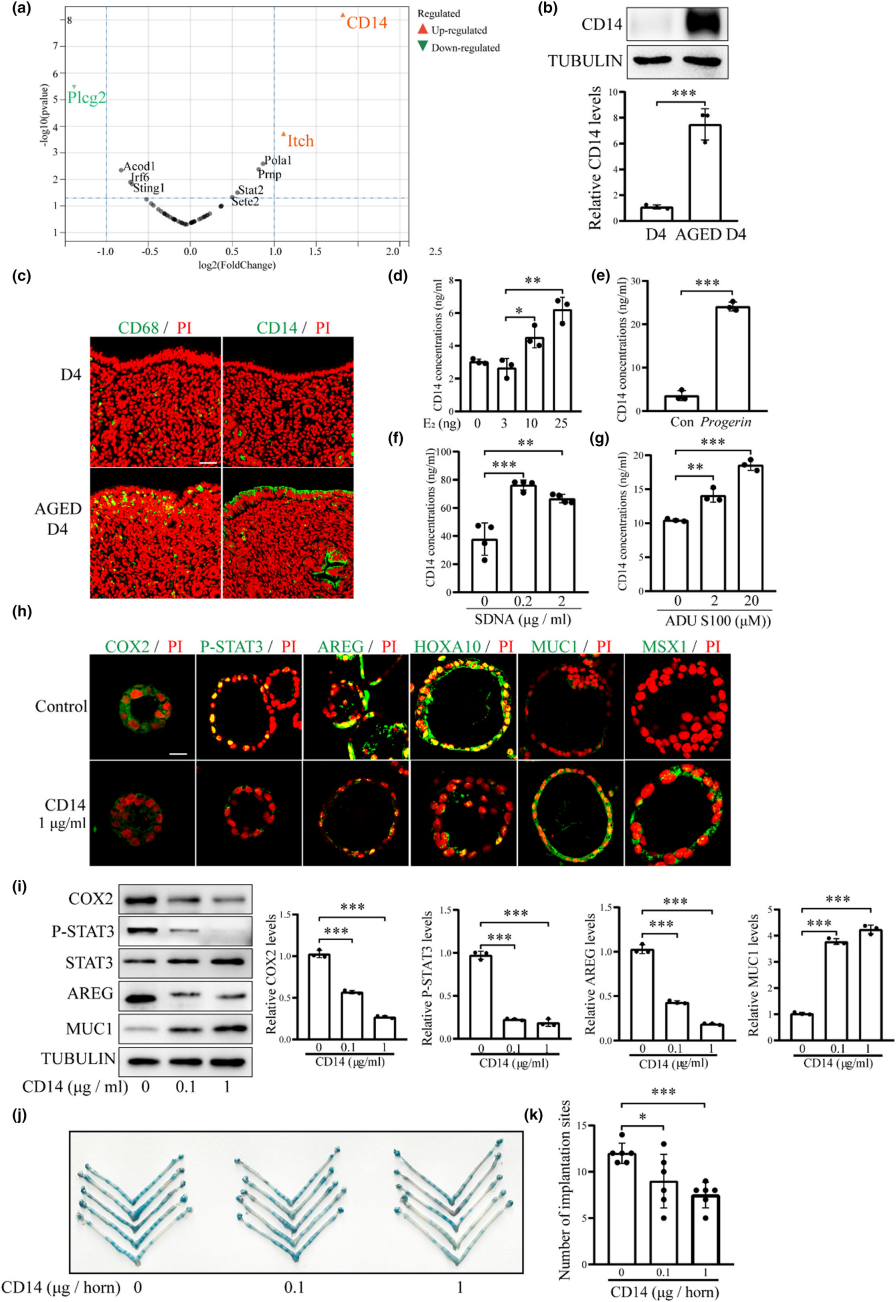
CD14 protein levels and its effects on uterine receptivity in aged mice. (a) Proteomic analysis of young (D4) and aged mouse (AGED D4) uteri on day 4 of pregnancy. (b) Western blot analysis of CD14 protein levels in young and aged mouse uteri on day 4 of pregnancy. (c) CD68 and CD14 immunofluorescence in young and aged mouse uteri on day 4 of pregnancy. Scale bar, 50 μm. (d) Uterine CD14 concentration after ovariectomized mice were injected subcutaneously with 0, 3, 10, or 25 ng E_2_ for 7 days. (e) CD14 concentration in the cultured medium after *Progerin* gene was overexpressed in mouse epithelial cells. Con, empty vector control; *Progerin*, *Progerin* overexpression. (f) CD14 concentration in the cultured medium after mouse epithelial cells were transfected with SDNA for 48 h. (g) CD14 concentration in the cultured medium after mouse epithelial cells were treated with ADU S100 for 48 h. (h) Immunofluorescence of the receptivity‐related proteins after mouse epithelial organoids were treated with CD14 for 48 h. Scale bar, 100 μm. (i) Western blot analysis of the receptivity‐related proteins after mouse epithelial organoids were treated with CD14 for 48 h. (j) A representative photograph showing the number of implantation sites on day 5 after recombinant CD14 was injected into the uterine lumen on day 4 of pregnancy. (k) Statistical analysis on the number of implantation sites on day 5 after recombinant CD14 was injected into the uterine lumen on day 4 of pregnancy. *p < 0.05; **, *p* < 0.01; ***p < 0.001.

Our data indicated the activation of cGAS‐STING pathway and abnormality of endometrial receptivity in aged mouse uterus. To further explore how the downstream targets of cGAS‐STING pathway execute the detrimental effects on endometrial receptivity, label‐free proteomic analysis was used to screen protein changes in aged mouse uterus. By comparing the downstream target genes of type I interferon with the human protein atlas database, a total of 55 proteins were differentially regulated in aged mouse uterus, of which 2 proteins were significantly upregulated and 1 protein was significantly downregulated (the fold difference was more than 2). Monocyte differentiation antigen CD14 (CD14) was the most significantly increased protein in aged mouse uterus (Figure 7a). Therefore, the expression and function of CD14 were further examined. Both immunofluorescence and Western blot showed that the protein levels of CD14 was significantly increased in aged mouse uterus (Figure 7b,c). CD14 is a coreceptor for TLR‐initiated proinflammatory responses in innate immune cells, particularly macrophages. CD14 glycoprotein receptor has been shown to promote macrophage activation (Sharygin et al., 2023, Rudnik et al., 2021). In our study, the immunofluorescence of CD68, a marker of macrophage, was stronger in the uterus of aging mice (Figure 7c). The protein level of CD14 in the uteri were significantly increased after ovariectomized mice were injected with 10 ng or 25 ng estradiol‐17β per mouse (Figure 7d). *Progerin* overexpression in mouse epithelial cells also stimulated CD14 secretion (Figure 7e). Similarly, CD14 secretion from mouse epithelial cells were also induced by STING agonist ADU S100 or SDNA (Figure 7f,g). The results indicated that PROGERIN could promote CD14 secretion from uterine epithelial cells via accumulation of cytoplasmic DNA and activation of CGAS‐STING pathway.

**FIGURE 8:**
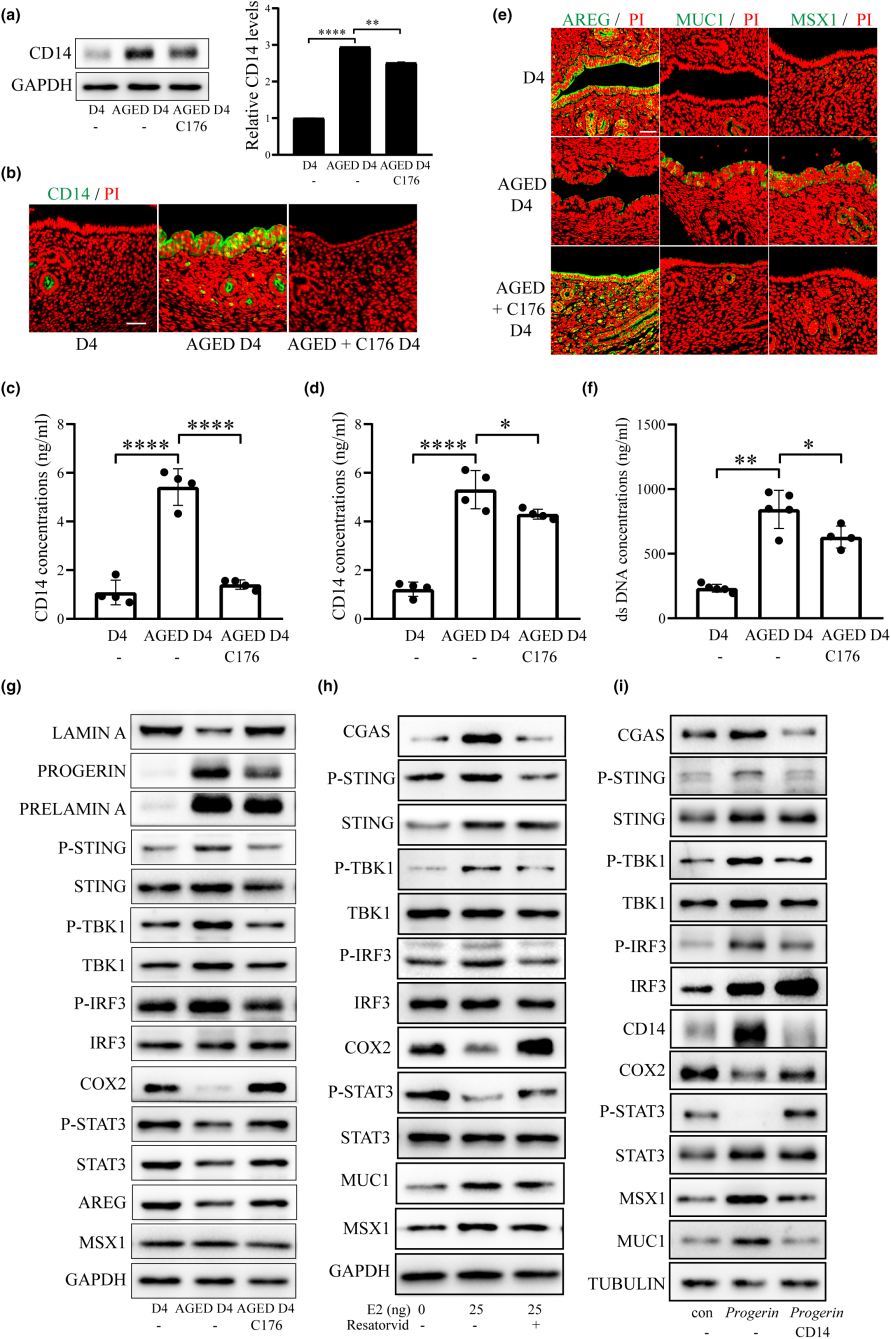
Effects of STING inhibitor (C176) on uterine receptivity and cGAS‐STING pathway. (a) Uterine CD14 protein levels young mice (D4), aged mice (AGED D4), and C176‐treated mice on day 4 of pregnancy. (b)CD14 immunofluorescence in young mice, aged mice, and C176‐treated mice on day 4 of pregnancy. Scale bar, 50 μm.(c) CD14 concentration in uteri of aged mice and C176‐treated mice. (d) CD14 concentration in serum of aged mice and C176‐treated mice. (e)Immunofluorescence of uterine AREG, MUC1 and MSX1 in young mice, aged mice, and C176‐treated mice. Scale bar, 50 μm. (f) Cytoplasmic dsDNA concentration in the uterus of young mice, aged mice, and C176‐treated mice. (g) Western blot analysis of the cGAS‐STING related proteins and receptivity‐related proteins in young mice, aged mice, and C176‐treated mice. (h) The protein levels CGAS‐STING pathway and uterine receptivity marker in mice on the D3 of pregnancy were injected with E2 (25 ng) and TLR4 inhibitor Resatorvid (10 mg/mL) subcutaneously for 24 h. (i) The protein levels CGAS‐STING pathway and uterine receptivity marker after epithelial organoids were overexpression of PROGERIN for 48 hr in the absence or presence of CD14 antibody. con, empty vector control; *Progerin*, *Progerin* overexpression. *, p < 0.05; **, p < 0.01; ****, p < 0.0001.

When the epithelial organoids were treated with CD14, the protein levels of uterine receptivity markers (COX2, HOXA10, P‐STAT3 and AREG) were significantly downregulated, while MUC1 and MSX1 were significantly upregulated (Figure 7h,i). After either 0.1 μg or 1 μg recombinant CD14 protein was injected into the uterine lumen on day 4 of pregnancy, the number of implantation sites was significantly decreased on day 5 of pregnancy (Figure 7j,k). The protein level of CD14 in aged mouse uterus was significantly reduced after aged mice were treated with C176, an inhibitor of STING (Figure 8a–c). CD14 concentration in serum also showed a similar change as uterine CD14 in aged mice (Figure 8d). Immunofluorescence and Western blot also indicated that treatment of aged mice caused an increase of AREG and a decrease of MUC1 and MSX1 (Figure 8e,g). The cytoplasmic concentration of ds DNA in aged mouse uterus was significantly reduced after aged mice were treated with C176, an inhibitor of STING (Figure 8f). Western blot showed that the protein level of LAMIN A was significantly increased, while protein levels of PROGERIN, PRELAMIN A, P‐STING, P‐TBK1 and P‐IRF3 were significantly decreased after aged mice were treated with C176 (Figure 8g). Treatments of mice on day 3 of pregnancy with E_2_ (25 ng/mouse) for 24 h could activate cGAS‐STING pathway and impair uterine receptivity, which was rescued by subcutaneous injection of Resatorvid (10 mg/mL), a specific inhibitor of TLR4 (Figure 8h). Additionally, *Progerin* overexpression in mouse uterine epithelial cells or uterine epithelial organoids also stimulated CGAS‐STING pathway and reduced the uterine receptivity, which was partially rescued by treatment with CD14 neutralization antibody (Figure 8i). These results indicated that PROGERIN‐induced activation of cGAS‐STING pathway and CD14 secretion could be partially rescued.

## 3 DISCUSSION

In our study, the increase in uterine PROGERIN in aged mice leads to the accumulation of cytoplasmic DNA and activation cGAS‐STING pathway. CD14 secretion from aged mouse uterus causes impaired uterine receptivity. Maternal gestational age may have negative consequences on the fetus as the number of surviving offspring drops in older females and embryo loss occurs after implantation (Tatone et al., 2008). Research has shown a notable decrease in endometrial receptivity, resulting in decreased delivery rates (from 46% to 21%) and elevated abortion percentages (from 17% to 41%), as the recipient's age exceeds 40 (Meldrum, 1993). The aging uterus may lead to increased embryo loss and decreased embryo development capacity at the implantation stage (Morton et al., 2017). Furthermore, in vitro induced decidualization of primary aged stromal cells reveals an association with defective stromal cell proliferation, subsequently affecting decidualization, endometrial receptivity, thickness, and embryo attachment (Berdiaki et al., 2022).

Increased PROGERIN results in altered nuclear shape and decreased functionality (Yang et al., 2005). PROGERIN‐caused aging shares important similarities with physiological senescence (Macicior & Ortega‐Gutiérrez, 2023). However, the relevance of PROGERIN in physiological aging during pregnancy remains unknown. In this study, PROGERIN level is significantly increased in aged mouse uterus. PROGERIN overexpression could also stimulate cytoplasmic DNA accumulation and cGAS‐STING activation. PRELAMIN A is also involved in physiological aging, showing similar toxic effects to PROGERIN and the overexpression of PRELAMIN A accelerates aging (Ragnauth et al., 2010). In our study, PRELAMIN A level is also significantly higher than that in control mice. ZMPSTE24 is responsible for the mature LAMIN A production from PRELAMIN A (Scaffidi & Misteli, 2006). Mutation in the zmpste24 gene is linked to aging (Shackleton et al., 2005). The inhibitors for ZMPSTE24 can improve the progeroid phenotypes of Zmpste24‐deficient mice (Fong et al., 2006). However, ZMPSTE24 level is obviously increased in aged mouse uterus. A previous study indicated that PRELAMIN A mutation increases PROGERIN formation, and the lack of a zmpste24 binding site on PROGERIN also leads to ZMPSTE24 increase (Messner et al., 2020). The LAMIN A, LAMIN B1 and LAMIN B2 are essential to maintain the integrity of nuclear envelope (Turgay & Medalia, 2017). In our study, the protein levels of LAMIN A, LAMIN B1 and LAMIN B2 are also significantly decreased. It has recently been revealed that the interaction of laminin a with Histone H3 is an important contributor to nuclear morphology, but PROGERIN inhibits lamina‐histone H3 interaction, which is related to nuclear morphological changes during aging (Schibler et al., 2023). A decrease in LAMIN A expression causes premature ageing syndrome (Karoutas & Akhtar, 2021). Loss of LAMIN B1 expression may also lead to premature aging (Freund et al., 2012).

In our study, E_2_ levels in serum are dramatically lowered in aged mice. Because of the nearly total suppression of ovarian function throughout aging, the secretion of hormones is modestly reduced except for gonadotropins (Arlt & Hewison, 2004). There is an aberrant uterine hormonal response in aged mice and the implantation and decidualization are significantly decreased in aged mice (Li et al., 2017). A mouse model of Polycystic ovary syndrome also has reduced serum E_2_ (Gu et al., 2023). In this study, the high level of uterine E_2_ in aged mice may be caused by the increase of E_2_ synthetase CYP19A1 and the decrease of E_2_ metabolizing enzyme HSD17B2. Increased E_2_ levels stimulate the proliferation of uterine epithelial cells. On day 4 of pregnancy, mouse uterine epithelial cells stop proliferating and begin to differentiate into the receptive phase (Li et al., 2011). Treatment with a high level of E_2_ causes cytoplasmic DNA accumulation and cGAS‐STING activation. A proper level of E_2_ is required for rodent embryo implantation (Yoshinaga & Adams, 1966). Either a low or high level of E_2_ is harmful for mouse uterine receptivity (Ma et al., 2003). We found that PROGERIN level is significantly upregulated by exogenous high level of E_2_. In both uterine Gp130‐and Stat3‐deficient mice, the increase in uterine E_2_ response leads to implantation failure (Sun et al., 2013). The uterine conditional deletion of ALK3 in mice also show abnormal embryo implantation and increased uterine E_2_ response (Monsivais et al., 2016). Therefore, our results suggest that excess E_2_ (10 or 25 ng/mouse) induces increased PROGERIN and activation of cGAS‐STING pathway. Bispenol A (BPA) with E_2_ic activity has adverse effects on embryo implantation and decidualization in early pregnancy (Jin et al., 2022).

Based on subcellular fraction, we showed that cytoplasmic DNA accumulation in aged mouse uterus is from leaked nuclear DNA, rather than mitochondrial DNA. In health cells, DNA located in the nucleus or mitochondria. Extracellular or cellular DNase enzymes, including DNase I, DNase II and TREX1, can digest leaked dsDNA to avoid activating cGAS‐STING pathway (Du et al., 2023). In our study, DNase I, DNase II and TREX1 are reduced in aged mouse uterus. Foreign DNAs are able to activate cGAS‐STING pathway in cultured epithelial cells. Moderate inflammation is required for a successful pregnancy, but excessive inflammation can lead to adverse pregnancy outcomes (Zhao et al., 2021). High mobility group box1 (HMGB1), ATP, uric acid and cell‐free fetal DNA are members of damage‐associated molecular pattern family. Recent studies indicate that ATP, HMGB1 and uric acid are involved in mouse decidualization (Li et al., 2023). However, high level of serum uric acid in preeclampsia results in abnormal pregnancy outcomes (Powers et al., 2006). Multiple factors can contribute to age‐related inflammation (Coppé et al., 2010). Inflammation is a hallmark of aging in the brain and inner ear (Watson et al., 2017). cGAS‐STING pathway is essential in the process of cell senescence (Li & Chen, 2018). Inflammatory immune response is involved in adenomyosis. The cGAS‐STING pathway may be activated in the tissues of patients with adenomyosis, while patients with adenomyosis usually suffer subfertility (Lin et al., 2021). Our results showed that nuclear DNA leakage in aged uterus leads to excessive inflammatory response and adverse pregnancy outcome. Treatment of aged mice with Sting inhibitor can rescue the abnormality on uterine receptivity.

cGAS‐STING activation finally promotes the production of type I interferons (Hopfner & Hornung, 2020). Our proteomic analysis showed that CD14 is obviously increased in aged mouse uterus. CD4 level is increased by treatment with foreign DNA, PROGERIN or excess E_2_. CD14 is regulated by type I interferon (Wu et al., 2019). CD14 is often recognized as a proinflammatory marker (Sánchez‐Cabo et al., 2023). Soluble CD14 level in saliva of patient's chronic periodontitis is proved to rise (Lappin et al., 2011). During aging, the concentration of soluble CD14 also increases (Staller et al., 2020). Increased maternal CD14 is associated with multiple adverse pregnancy outcomes (Manousopoulou et al., 2020). Our results also demonstrated that embryo implantation is inhibited by intraluminal CD14 injection and uterine receptivity is adversely affected by CD14.

## 4 CONCLUSION

In aged mouse uterus, there is an obvious increase of cytoplasmic DNA, PROGERIN and E_2_ activity. The activation of cGAS‐STING pathway in aged uterus leads to CD14 secretion and the abnormality of embryo implantation.

## 5 MATERIALS AND METHODS

### 5.1 Animals and treatments

ICR mice (6–8 weeks old) were bought from Hunan Slaike Jingda Laboratory Animal Co. and housed in a temperature‐controlled environment with a 12 h photoperiod. All the mouse experiments were approved by the Animal Use and Care Committee of South China Agricultural University. In group 1, 6–8 weeks‐old female mice were served as control mice (young mice). In group 2, female mice were maintained to 12‐months old to undergo natural aging. In group 3, 11‐months‐old female mice were intraperitoneally administrated daily with STING inhibitor (C176, 10 μM, HY‐112906, MedChemExpress, NJ, USA) to rescue aging. Female mice in each group were mated with 8–12 weeks old male mice to induce pregnancy (day 1 is the day of vaginal plug). The vasectomized male mice were used for pseudopregnancy. Implantation sites were identified by intravenous injection of 0.1 mL of 1% Chicago blue dye (Sigma‐Aldrich, St. Louis, MO) dissolved in saline.

Female mice (6–8 weeks old) were ovariectomized and subcutaneously injected with different concentrations of estradiol‐17β (HY‐B0141, MedChemExpress) dissolved in sesame oil (3, 10, 25, or 100 ng per mouse) (*N* = 5 mice). Sesame oil was used as control. Mice were sacrificed to collect uteri for further analysis.

### 5.2 Immunofluorescence

Immunofluoresence was performed as described previously (Chen et al., 2023). Uterine tissues were fixed in 10% PBS‐buffered formalin and paraffin‐embedded. After paraffin sections (5 μm) were dewaxed and rehydrated, antigen retrieval was performed in EDTA or citric acid solution. After blocking with 10% horse serum, sections were incubated with each primary antibody at 4°C overnight. The primary antibodies used in this study included anti‐PROGERIN (1:200, ab66587, Abcam, Cambridge, UK), anti‐LAMIN A (1:200, ab26300, Abcam), anti‐H3K9ME3 (1:200, ab8898, Abcam), anti‐ds DNA IgG‐ (1:200, sc‐58,749, Santa cruz, Dallas, TX), anti‐dsDNA IgM‐(1:200, AC‐30‐10, BIO‐RAD), anti‐MSX1 (1:100, BS‐8512R, Bioss, Beijing, China), anti‐COX2 (1:400, 12,282 T, Cell Signaling Technology, Danvers, MA), anti‐MUC1 (1:100, Santa Cruz), anti‐HOXA10 (1:100, Sc‐28,602, Santa Cruz), anti‐HB‐EGF (1:100, A1695, Abclonal, Wuhan, China), anti‐p‐STAT3 (1:200, 9131S, Cell Signaling Technology), anti‐AREG (1:100, BS‐3847R, BIOSS), and anti‐CYP19A1 (1:200, PA1‐21398, Invitrogen, Carlsbad, CA). After three washes with PBS, the sections were incubated with matched secondary antibodies (2.5 μg/mL, G21234, Invitrogen, Carlsbad, CA) for 30 min at 37°C, counterstained with 4, 6‐diamidino‐2‐phenylindole dihydrochloride (20 g/mL, DAPI, D9542, Sigma‐Aldrich) or propidium iodide (5 g/mL, PI, P4170, Sigma‐Aldrich), and mounted with ProLong Diamond Antifade Mountant (Thermo Fisher's, Waltham, MA). Laser scanning confocal microscopy (Leica, Germany) was used to collect the images.

### 5.3 Immunohistochemistry

Immunohistochemistry was performed as described previously (Chen et al., 2023). In brief, paraffin sections (5 μm) were deparaffinized, rehydrated, and antigen‐retrieved in 10 mM citrate buffer for 10 min. Endogenous horseradish peroxidase (HRP) activity was inhibited by 3% H_2_O_2_ solution in methanol. After washing three times with PBS, sections were blocked in 10% horse serum at 37°C for 1 h and incubated in each primary antibody overnight at 4°C. Primary antibodies used in this study included anti‐KI67 (1:200, GB111141, Exilon, Guangzhou), anti‐HAND2 (1:200, sc‐9409, Santa Cruz), anti‐ERα (1:200, ab32063, Abcam), and anti‐P‐ERα (1:200, SAB4504399, Sigma‐Aldrich, St. Louis, MO). After washing the sections, sections were incubated with matched biotinylated secondary antibody (1:200, Zhongshan Jinqiao, Beijing, China) and streptavidin‐horseradish peroxidase complex (1:200, Zhongshan Jinqiao), respectively. Positive signals were visualized using DAB horseradish peroxidase chromogenic kit (Zhongshan Jinqiao). The nuclei were counterstained with hematoxylin. Each experiment was repeated at least three times.

### 5.4 Measurement of DNA

Cell fraction was performed as previously described (Antón & Traba, 2022). Tissues were homogenized in digitonin buffer, transferred to 1.5 mL centrifuge tubes, incubated at 4°C on a shaker for 10 min, and centrifuged at 1000× g for 10 min at 4°C. The supernatant was collected and centrifuged again at 10,000× g at 4°C for 30 min to get pure cytosolic fraction. DNeasy Blood & Tissue Kit (D3096‐01, Omega) was used to purify DNA from the cytosolic fractions. DNAs were eluted from MicroElute DNA Mini column and store at −20°C for further analysis.

The concentration of dsDNA in the pure cytosolic fraction was measured using the dsDNA Quantification kit (P7589, Invitrogen). The fluorescence in the samples was analyzed using a fluorescence microplate reader at fluorescence wavelengths (excitation ~480 nm, emission ~520 nm) and calculated through a dsDNA standard curve.

### 5.5 Measurement of E_2_ concentration

After mouse blood was collected in anticoagulant tubes, serum was separated through centrifugation. Uterine tissues were homogenized to collect the supernatant through centrifugation. Protein concentration was measured to standardize each sample. E_2_ concentration in serum or uterine supernatants was measured by mouse E_2_ ELISA Kit (CSB‐E07280m, Cusabio, Wuhan, China) in triplicate according to the manufacturer's protocol.

### 5.6 Measurement of mouse CD14 protein level

Mouse uterus was homogenized and centrifuged to collect the supernatants. The cultured medium was collected from cultured mouse epithelial cells. A mouse CD14 ELISA Kit (EM0039, Wuhan Fine Biotech, Wuhan, China) was used for the analysis. CD14 concentration quantification was performed in triplicate according to the manufacturer's protocol, readings were taken at 450 nm with a Molecular Device Spectra Max M5 instrument and calculated using a CD14 standard curve.

### 5.7 Isolation and treatment of mouse uterine luminal epithelial cells

Uterine luminal epithelial cells were isolated as previously described (Chen et al., 2023). Mouse uteri from day 4 pseudopregnant mice was longitudinally cut, rinsed in HBSS for three times and incubated in the digestion solution (0.2% trypsin, 6 mg/mL Dispase, 4.3 mL HBSS, and 50 μL streptomycin/penicillin) at 4°C for 1.5 h, room temperature for 30 min, and 37°C for 10 min. Cells were rinsed three times in HBSS and cultured in DMEM/F‐12 medium with 10% heat‐inactivated fetal bovine serum (FBS) for 30 min. The unattached epithelial cells were transferred into new culture plates precoated with ECM (1:100, E0282, Sigma‐Aldrich). Luminal epithelial cells were treated with ADU‐S100 (2 and 20 μM, HY‐12885A, MedChemExpress), Salmon SDNA (0.2 and 2 μg/mL) or CD14 (0.1 and 1 μg/mL, HY‐P75444, MedChemExpress) in DMEM/F12 with 2% charcoal‐treated FBS (cFBS, Biological Industries, Cromwell, CT).

### 5.8 Culture and treatment of mouse uterine luminal epithelial organoids

Uterine luminal epithelial organoids were prepared as previously described (Chen et al., 2023). Uteri from day 4 pseudopregnant mice was longitudinally cut, rinsed in HBSS for three times and digested in the digestion solution (100 mg/mL trypsin, 6 mg/mL Dispase, 3.5 mL HBSS, 50 μL streptomycin/penicillin) at 4°C for 1 h, room temperature for 1 h, and 37°C for 10 min, respectively. After rinsed three times in HBSS, the cells were collected through centrifugation at 1200 g for 5 min and suspended in DMEM/F12 medium at the density of 1.5 × 10^7^ cells/ml. The epithelial cell suspension was mixed with ice‐precooled ECM (1:3, 356,231, BD biocoat, Becton‐Dickinson, MA), seeded onto the preheated 24‐well plates and cultured in organoid medium.

### 5.9 Western blot

Western blot was performed as previously described (Chen et al., 2023). After protein samples were separated through PAGE gels and transferred onto PVDF membranes, the membranes were blocked in 5% nonfat milk, and incubated with each primary antibody and matched HRP‐conjugated secondary antibody (1:5000, Invitrogen) for 1 h. The signals were visualized with an ECL Chemiluminescent Kit (Millipore, USA). Each experiment was repeated at least three times. The primary antibodies used in this study included P16 (1:1000, CSB‐PA003618, CUSABIO), P21 (1:1000, 28,248‐1‐AP, Proteintech, Wuhan, China), P53 (1:1000, 60,283, Proteintech), LAMIN A (1:1000), LAMIN B1(1:1000, ab133741, Abcam), LAMIN B2 (1:1000, ab151735, Abcam), P‐STAT3 (1:1000), STAT3 (1:1000, 9139 s, Cell Signaling Technology), AREG (1:1000, BS‐3847R, Bioss), MUC1 (1:1000, ab45167, abcam), MSX1 (1:1000), ZMPSTE24 (1:1000, FNab09649, Wuhan Fine Biotech), PROGERIN (1:1000), DNASE1 (1:1000, ab224617, Abcam), DNASE2 (1:1000, BS‐23335R, Bioss), TREX1 (1:1000, ab300445, Abcam), cGAS (1:1000, 15,102 T, Cell Signaling Technology), P‐STING (1:1000, 50,907 T, Cell Signaling Technology), STING (1:1000, ab288157, Abcam), P‐TBK1 (1:500, 5483S, Cell Signaling Technology), TBK1 (1:1000, 38066S, Cell Signaling Technology), P‐IRF3 (1:1000, 29047S, Cell Signaling Technology), IRF3 (1:1000, 4302S, Cell Signaling Technology), P‐P65 (1:1000, 3033S, Cell Signaling Technology), COX‐2 (1:1000, 12,282 T, Cell Signaling Technology), PRELAMIN A (1:1000, MABT858, Sigma‐Aldrich), CD14 (ab221678, Abcam), TUBULIN (1:1000, 2144 S, Cell Signaling Technology), and GAPDH (1:1000, sc‐32,233, Santa Cruz Biotechnology).

### 5.10 Transfection of overexpression plasmids

*Progerin* overexpression plasmid was purchased from Feng Hui Biotechnology Co., LTD. After *Progerin* cDNA plasmid was amplified and extracted with the Endo‐Free Plasmid Maxi Kit (OMEGA, D6926), 1 μg *Progerin* plasmid or empty vector control was transfected into mouse uterine epithelial cells or uterine epithelial organoids with Lipo2000 (11,668,019, Invitrogen) for 6 h and cultured in DMEM/F12 with 10% cFBS for 24 or 48 h.

### 5.11 Proteomic analysis

After myometrium was removed from mouse uteri, the remaining endometrium was pooled from three mice and used for Label‐free quantitative proteomic analysis by Novogene (Beijing, China). Proteome Discoverer was used to calculate relative quantitative values for each protein. The relative quantitative value of each protein in two comparison samples was tested by significance test. *P* value for the significance was ≤0.05.

### 5.12 Realtime RT‐PCR

The total RNA was extracted using the Trizol Reagent Kit (9109, Takara, Japan), digested with RQ1 deoxyribonuclease I (Promega, Fitchburg, WI), and reverse‐transcribed into cDNA with the Prime Script Reverse Transcriptase Reagent Kit (Takara, Japan). For realtime PCR, the cDNA was amplified using a SYBR Premix Ex Taq Kit (TaKaRa) on the CFX96 Touch Realtime System (Bio‐Rad). Data were analyzed using the 2^‐△△^Ct method and normalized to mouse Rpl7 level. Table 1 showed the corresponding primer sequences for each gene. Every experiment was carried out at least three times.

**TABLE 1:** Primers sequences used in this study.

Gene	Species	Sequence (5′‐3′)	Application	Accession number	Product size
*16S*	Mouse	CACTGCCTGCCCAGTGA ATACCGCGGCCGTTAAA	RT‐QPCR		
*β‐globin*	Mouse	GGTGCTGACTGCTTTTGGAG TTGCCGAAGTGACTAGCCAAA	RT‐QPCR		162 bp
*Areg*	Mouse	CACAGCGAGGATGACAAGGA GATAACGATGCCGATGCCAATA	RT‐QPCR	NM_001277266.1	102 bp
*C3*	Mouse	TGGACCAGACCGAACAGT GAAGGCAGCATAGGCAGA	RT‐QPCR	NM_009778.3	125 bp
*Dloop*		Mouse	AATCTACCATCCTCCGTGAAACC TCAGTTTAGCTACCCCCAAGTTTAA	RT‐QPCR	
*Hsd11b2*	Mouse	CGTTTGCCTCTGAGTCAGCAT AAGCAGGCCACGGATAAGAG	RT‐QPCR	NM_008290.2	190 bp
*Ihh*	Mouse	TGCTGTCAATGGGCGGA CCTTCCCCAGTCCCAGGTAG	RT‐QPCR		
*Ltf*	Mouse	AGCCAACAAATGTGCCTCTTC CCTCAAATACCGTGCTTCCTC	RT‐QPCR	NM_008522	119 bp
*Nd4*	Mouse	AACGGATCCACAGCCGTA AGTCCTCGGGCCATGATT	RT‐QPCR		
*Progerin*	Mouse	GCAACAAGTCCAATGAGGACCA CATGATGCTGCAGTTCTGGGGGCTCTGGAC	RT‐QPCR		
*Rpl7*	Mouse	GCAGATGTACCGCACTGAGATTC ACCTTTGGGCTTACTCCATTGATA	RT‐QPCR	NM_011291.5	129 bp

### 5.13 Statistical analysis

The data were analyzed using GraphPad Prism8.0. Student's T test was used to examine the differences between two groups. One‐ or two‐way analysis of variance (ANOVA) test was used to compare multiple groups. All the experiments were done at least three times independently. There were at least three mice per group. The data were presented as mean standard deviation (SD). A p value of less than 0.05 was considered as statistically significant.

## AUTHOR CONTRIBUTIONS

Si‐Ting Chen, Conceptualization, Data curation, Formal analysis, Investigation, Methodology, Writing—original draft; Feng Ran, Wen‐Wen Shi, and Cheng‐Kan Liu, Data curation, Investigation, Validation; Hui‐Na Luo, Li‐Juan Wu, Ying Wu, and Tong‐Tong Zhang, Data curation, Formal analysis, Investigation; Zeng‐Ming Yang, Conceptualization, Supervision, Funding acquisition, Writing—original draft, Project administration, Writing—review and editing.

## FUNDING INFORMATION

This study was supported by National Natural Science Foundation of China (32370915; 32171114).

## CONFLICT OF INTEREST STATEMENT

The authors declare that they have no conflict of interest.

## ETHICS STATEMENT

All animal protocols were approved by the Animal Care and Use Committee of South China Agricultural University.

## CONSENT

N/A.

## Data Availability

All data needed to evaluate the conclusions in the paper are presented in the paper. All materials in this manuscript are available from the corresponding author on reasonable request.
