# Correction to “The Activation of cGAS‐STING Pathway Causes Abnormal Uterine Receptivity in Aged Mice”

**DOI:** 10.1111/acel.70536

**Published:** 2026-05-09

Chen, S. T., W. W. Shi, F. Ran, et al. 2024. “The Activation of cGAS‐STING Pathway Causes Abnormal Uterine Receptivity in Aged Mice.” *Aging Cell* 23: e14303. https://doi.org/10.1111/acel.14303.

In the published version of this article, several errors occurred during the assembly of Figures 5a, 7b, 8g, and 8i.

In Figure 5a, an incorrect representative Western blot band was inadvertently included for the P‐STING. Additionally, the histogram for P‐IRF3 was inadvertently misplaced during figure assembly. The corrected figure is shown below.

**FIGURE 5:**
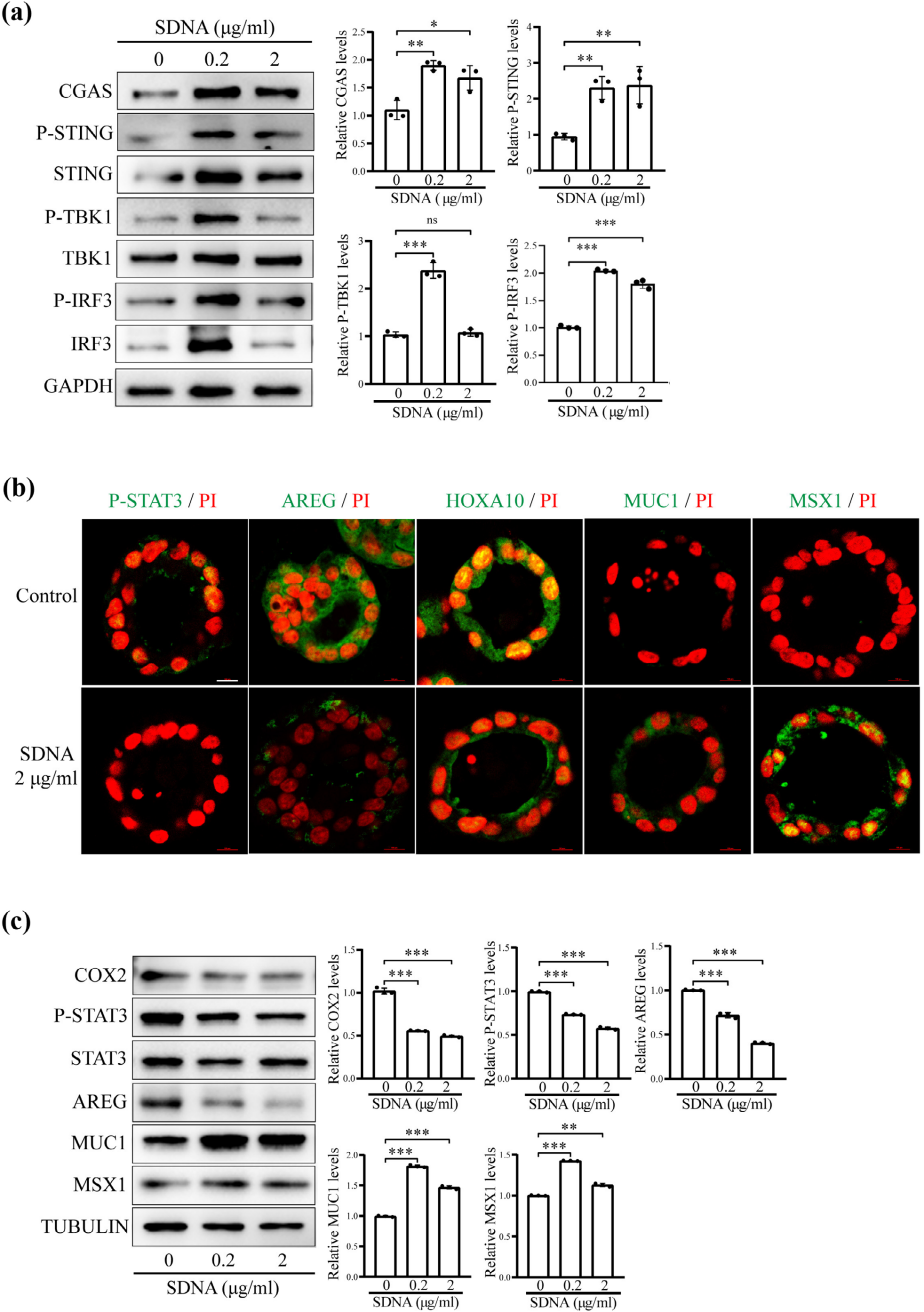
Foreign DNA activates cGAS‐STING pathway and affects uterine receptivity. (a) Western blot analysis of cGAS‐STING‐related protein levels after mouse epithelial cells were transfected with salmon sperm DNA (SDNA) for 48 h. (b) Immunofluorescence of p‐STAT3, AREG, HOXA10, MUC1, and MSX1 after mouse epithelial organoids were transfected treated with SDNA for 48 h. Scale bar, 100 μm. (c) Western blot analysis of receptivity‐related protein levels after mouse epithelial organoids were transfected with SDNA for 48 h. ns, not significant; *p < 0.05; **p < 0.01; ***p < 0.001.

In Figure 7b, an incorrect representative Western blot band was inadvertently included for the TUBULIN. The corrected figure is shown below.

**FIGURE 7:**
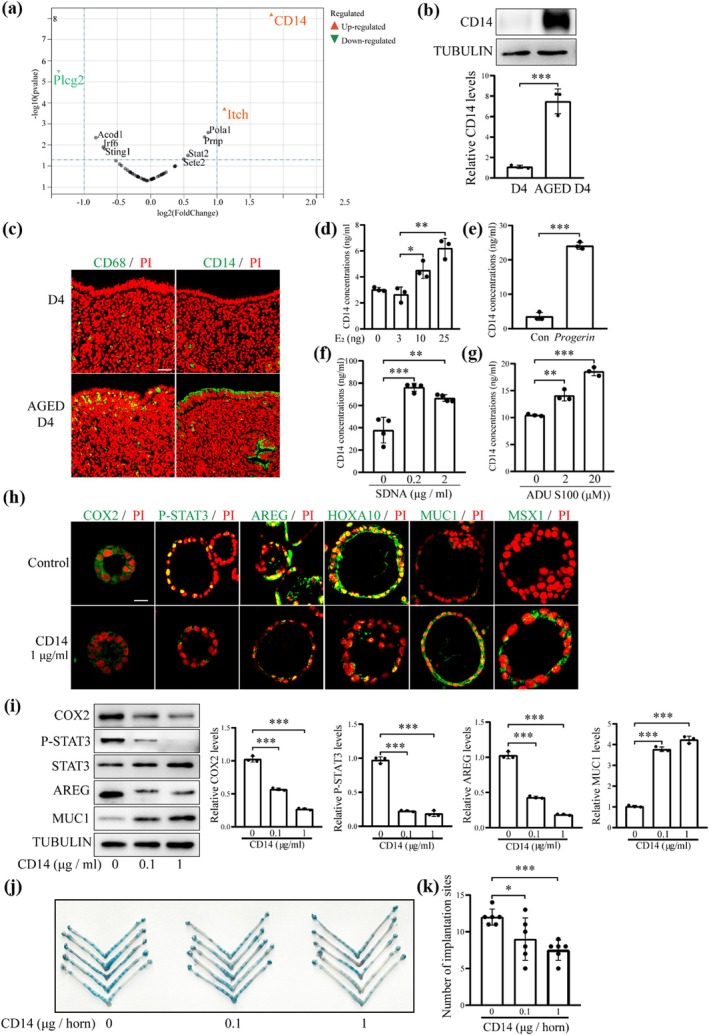
CD14 protein levels and its effects on uterine receptivity in aged mice. (a) Proteomic analysis of young (D4) and aged mouse (AGED D4) uteri on day 4 of pregnancy. (b) Western blot analysis of CD14 protein levels in young and aged mouse uteri on day 4 of pregnancy. (c) CD68 and CD14 immunofluorescence in young and aged mouse uteri on day 4 of pregnancy. Scale bar, 50 μm. (d) Uterine CD14 concentration after ovariectomized mice were injected subcutaneously with 0, 3, 10, or 25 ng E2 for 7 days. (e) CD14 concentration in the cultured medium after *Progerin* gene was overexpressed in mouse epithelial cells. Con, empty vector control; *Progerin*, *Progerin* overexpression. (f) CD14 concentration in the cultured medium after mouse epithelial cells were transfected with SDNA for 48 h. (g) CD14 concentration in the cultured medium after mouse epithelial cells were treated with ADU S100 for 48 h. (h) Immunofluorescence of the receptivity‐related proteins after mouse epithelial organoids were treated with CD14 for 48 h. Scale bar, 100 μm. (i) Western blot analysis of the receptivity‐related proteins after mouse epithelial organoids were treated with CD14 for 48 h. (j) A representative photograph showing the number of implantation sites on day 5 after recombinant CD14 was injected into the uterine lumen on day 4 of pregnancy. (k) Statistical analysis on the number of implantation sites on day 5 after recombinant CD14 was injected into the uterine lumen on day 4 of pregnancy. *p < 0.05; **p < 0.01; ***p < 0.001.

In Figure 8g, the STAT3 band for loading control is correct. However, the same band was inadvertently included as AERG band. Additionally, in Figure 8i, the STING band for loading control is correct. However, the same band in Figure 8i was inadvertently included as STAT3 for loading control. The corrected figure is shown below.

**FIGURE 8:**
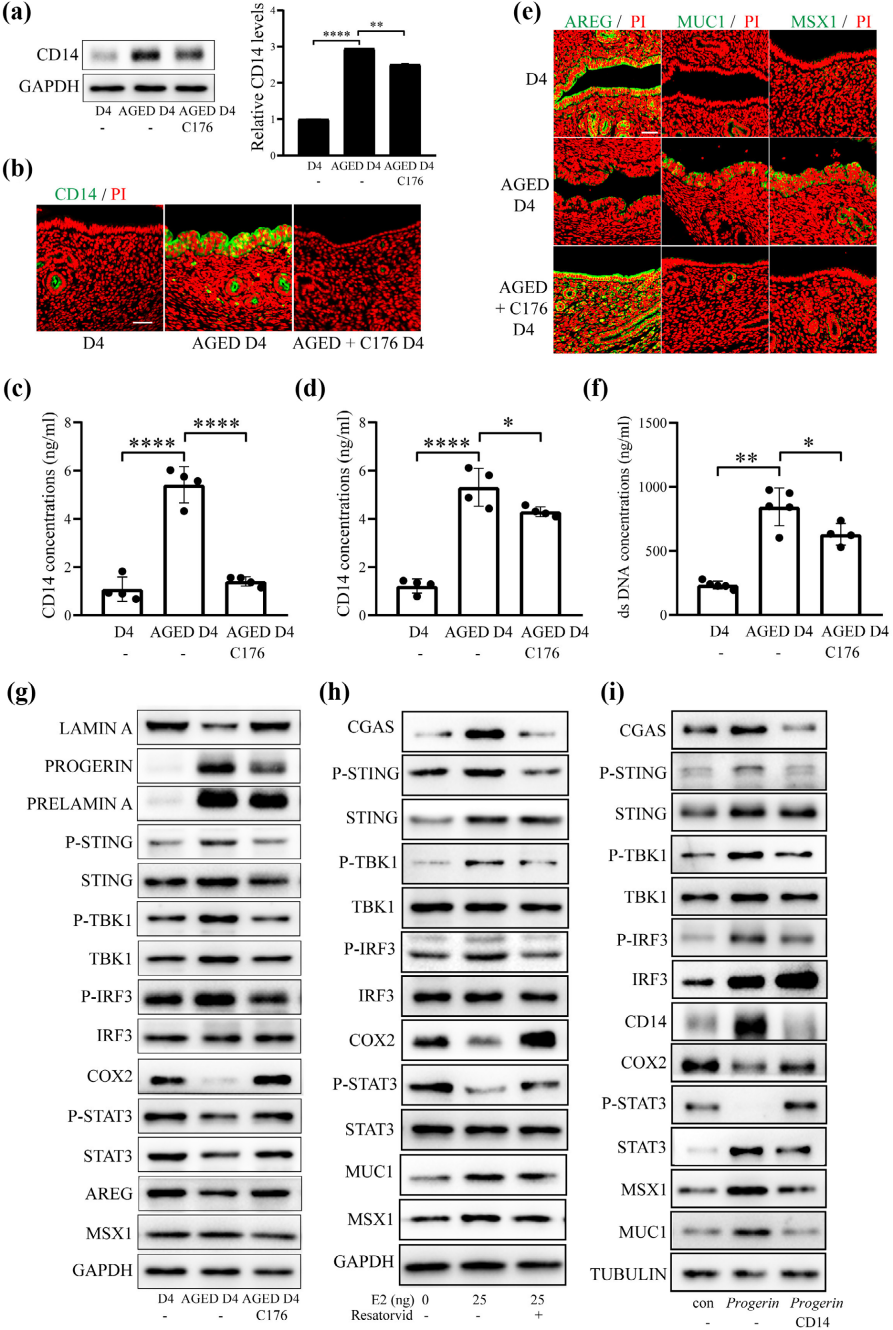
Effects of STING inhibitor (C176) on uterine receptivity and cGAS‐STING pathway. (a) Uterine CD14 protein levels young mice (D4), aged mice (AGED D4), and C176‐treated mice on day 4 of pregnancy. (b) CD14 immunofluorescence in young mice, aged mice, and C176‐treated mice on day 4 of pregnancy. Scale bar, 50 μm. (c) CD14 concentration in uteri of aged mice and C176‐treated mice. (d) CD14 concentration in serum of aged mice and C176‐treated mice. (e) Immunofluorescence of uterine AREG, MUC1, and MSX1 in young mice, aged mice, and C176‐treated mice. Scale bar, 50 μm. (f) Cytoplasmic dsDNA concentration in the uterus of young mice, aged mice, and C176‐treated mice. (g) Western blot analysis of the cGAS‐STING related proteins and receptivity‐related proteins in young mice, aged mice, and C176‐treated mice. (h) The protein levels CGAS‐STING pathway and uterine receptivity marker in mice on the D3 of pregnancy were injected with E2 (25 ng) and TLR4 inhibitor Resatorvid (10 mg/mL) subcutaneously for 24 h. (i) The protein levels CGAS‐STING pathway and uterine receptivity marker after epithelial organoids were overexpression of PROGERIN for 48 h in the absence or presence of CD14 antibody. Con, empty vector control; *Progerin*, *Progerin* overexpression. *p < 0.05; **p < 0.01; ****p < 0.0001.

The corrections reported are limited to the representative image presentation and do not affect the conclusions of the study.

We apologize for these errors.

